# The Expansion of Myeloid-Derived Suppressor Cells Correlates With the Severity of Pneumonia in Kidney Transplant Patients

**DOI:** 10.3389/fmed.2022.795392

**Published:** 2022-02-15

**Authors:** Bo Peng, Yulin Luo, Quan Zhuang, Junhui Li, Pengpeng Zhang, Min Yang, Yu Zhang, Gangcheng Kong, Ke Cheng, Yingzi Ming

**Affiliations:** ^1^Transplantation Center, The Third Xiangya Hospital, Central South University, Changsha, China; ^2^Engineering and Technology Research Center for Transplantation Medicine of National Health Commission, Changsha, China

**Keywords:** myeloid-derived suppressor cells (MDSCs), pneumonia, kidney transplantation, immune monitoring, immunosuppression

## Abstract

**Background:**

Pneumonia is one of the most frequent but serious infectious complications post kidney transplantation. Severe pneumonia induces sustained immunosuppression, but few parameters concerning immune status are used to assess the severity of pneumonia. Myeloid-derived suppressor cells (MDSCs) are induced under infection and have the strong immunosuppressive capacity, but the correlation between MDSCs and pneumonia in kidney transplant recipients (KTRs) is unknown.

**Methods:**

Peripheral blood MDSCs were longitudinally detected in 58 KTRs diagnosed with pneumonia using flow cytometry and in 29 stable KTRs as a control. The effectors of MDSCs were detected in the plasma. Spearman's rank correlation analysis was performed to determine the correlation between MDSCs and the severity of pneumonia as well as lymphopenia.

**Results:**

The frequency of MDSCs and effectors, including arginase-1, S100A8/A9, and S100A12, were significantly increased in the pneumonia group compared with the stable group. CD11b^+^CD14^+^HLA-DR^low/−^CD15^−^ monocytic-MDSCs (M-MDSCs) were higher in the pneumonia group but showed no significant difference between the severe and non-severe pneumonia subgroups. CD11b^+^CD14^−^CD15^+^ low-density granulocytic-MDSCs (G-MDSCs) were specifically increased in the severe pneumonia subgroup and correlated with the severity of pneumonia as well as lymphopenia. During the study period of 2 weeks, the frequencies of MDSCs and G-MDSCs were persistently increased in the severe pneumonia subgroup.

**Conclusions:**

MDSCs and G-MDSCs were persistently increased in KTRs with pneumonia. G-MDSCs were correlated with the severity of pneumonia and could thus be an indicator concerning immune status for assessing pneumonia severity.

## Introduction

Kidney transplantation has become the optimal treatment for patients with end-stage renal disease (ESRD). The long-term or even lifelong administration of immunosuppressive drugs remains essential for most kidney transplant recipients (KTRs) to prevent allograft rejection, but it dampens the host immune response to pathogens and increases the risk of infection at the same time (1). Compared with the general population, KTRs have a substantially higher requirement for admission and a higher mortality rate due to infectious diseases (2, 3). The cumulative infection incidence is as high as 78.0% at 5 years post-kidney transplantation (4). Although the mortality rate due to infections has declined in the current era, it is still the second most common cause of death for KTRs, only following cardiovascular death, according to the United States Renal Data System (1996–2014) (5). Post-transplant infection also contributes to the increase in death-censored graft failure (DCGF), indicating that infection is a severe complication post-kidney transplantation (4).

Pneumonia is one of the most frequent and serious infectious diseases for KTRs and is usually a co-infection caused by multiple pathogens, such as common bacteria, tuberculosis, and opportunistic pathogens, such as cytomegalovirus and *Pneumocystis jirovecii* (4, 6, 7). A notable characteristic of KTRs diagnosed with pneumonia is that patients are generally in a state of significant immunosuppression, manifesting consistent lymphopenia, and low immune response to pathogens even when withdrawing all of the immunosuppressive drugs (8). Our group previously found that, compared with stable KTRs, patients with pneumonia had significantly lower absolute cell counts of CD3^+^CD4^+^ T cells, CD3^+^CD8^+^ T cells, CD19^+^ B cells, and natural killer cells, as well as lower expression of human leukocyte antigen (HLA)-DR on monocytes but higher expression of CD64 on neutrophils (9). A composite equation with these immune biomarkers better assessed the association between immune status and pneumonia than any single parameter did and could predict the prognosis of pneumonia (9). However, the current criteria used to evaluate the severity of pneumonia, such as the Infectious Diseases Society of America/American Thoracic Society (IDSA/ATS) criteria for severe community-acquired pneumonia (CAP) (10), the CURB-65 score (11), the pneumonia severity index (PSI) (12) and the sequential organ failure assessment (SOFA) score (12), only focus on immunocompetent patients and involve few parameters to assess the immune status of the patients. Therefore, more parameters concerning immune status should be added for KTRs to accurately evaluate the severity of pneumonia.

During pneumonia, emergency myelopoiesis is induced, and the normal differentiation of immature myeloid cells into mature granulocytes and monocytes is blocked (13, 14). The heterogeneous populations of immature myeloid cells induced under pathological conditions have strong immunosuppressive properties and are termed “myeloid-derived suppressor cells” (MDSCs) (15). Two major subsets of MDSCs in humans have been identified: CD11b^+^CD14^−^CD15^+^ granulocytic-MDSCs (G-MDSCs) or polymorphonuclear-MDSCs (PMN-MDSCs) with a low density, and CD11b^+^CD14^+^HLA-DR^low/−^CD15^−^ monocytic-MDSCs (M-MDSCs) (16). Previously, the expansion of MDSCs has been reported in patients with sepsis or coronavirus disease 2019 (COVID-19), and it was correlated with the severity of the diseases (17–19). Our previous work also found a lower expression of HLA-DR on monocytes in KTRs with pneumonia, indicating the possibility of MDSC expansion in these patients (9). However, the detailed association between MDSCs and pneumonia in KTRs has not been elucidated.

In this research, we performed a prospective longitudinal analysis of peripheral blood MDSCs in KTRs and studied the association between MDSCs and the severity of pneumonia.

## Materials and Methods

### Study Design and Population

**Figure 1:**
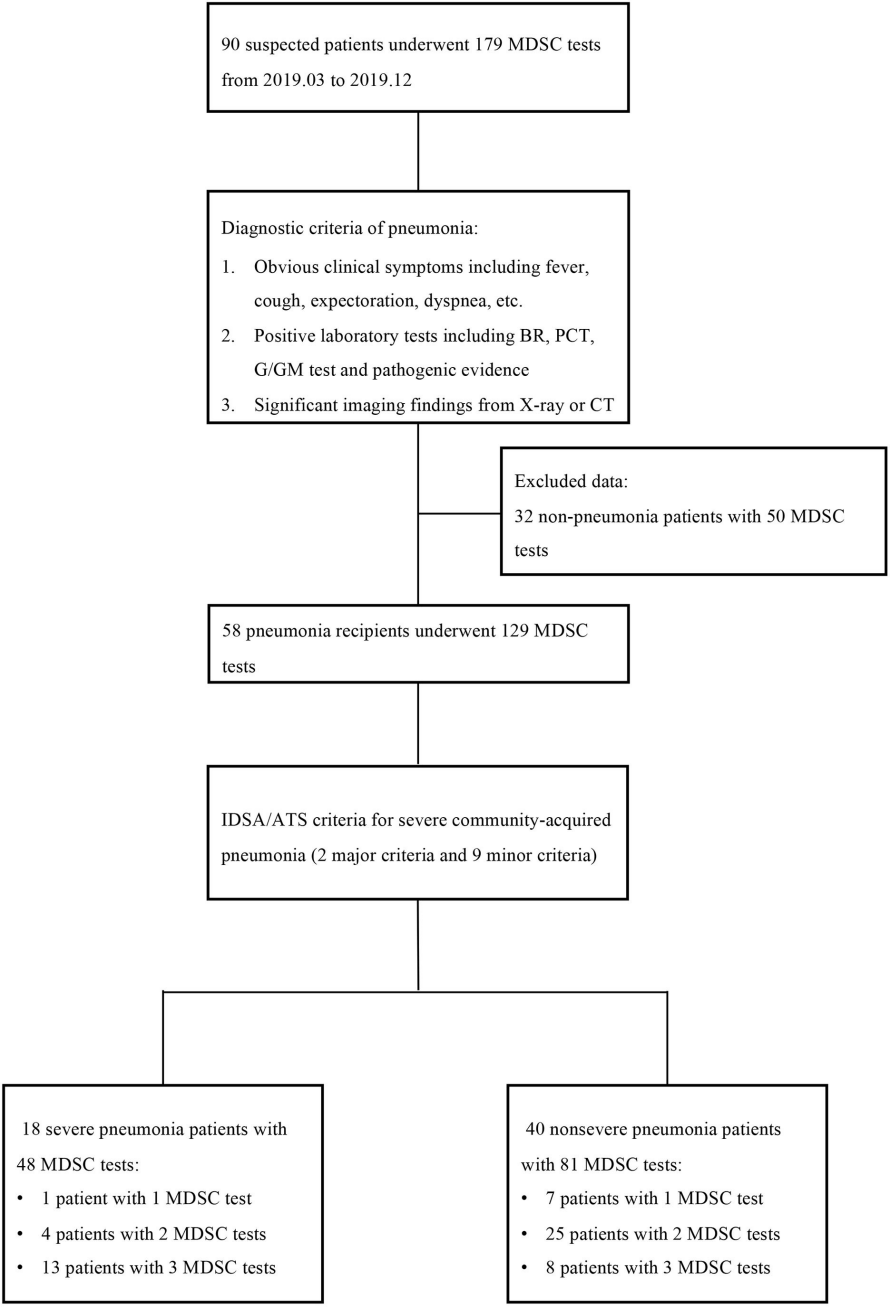
The study flow and diagnostic criteria of pneumonia. Ninety suspected patients with 179 myeloid-derived suppressor cell (MDSC) tests were first enrolled, but 32 patients with 50 tests were excluded according to the diagnostic criteria of pneumonia. The remaining 58 patients were further classified into the severe pneumonia subgroup and non-severe pneumonia subgroup according to the IDSA/ATS criteria. MDSC, myeloid-derived suppressor cell; BR, blood routine; PCT, procalcitonin; IDSA/ATS, infectious diseases society of america/american thoracic Society.

This was a prospective and observational study that longitudinally detected peripheral blood MDSCs in KTRs with pneumonia. KTRs who were suspected of pneumonia and admitted to the Transplantation Center, The Third Xiangya Hospital, Central South University from March 1, 2019, to December 31, 2019, were enrolled. Peripheral blood MDSCs were detected at three time points, namely, 1–3 days, 5–8 days, and 12–15 days post-admission. All the patients were 18–65 years old, and those who did not meet the diagnostic criteria of pneumonia were excluded (Figure 1). Another group of stable outpatient KTRs was also recruited as a control and received the MDSC test once. Informed consent was obtained for each patient, and the study was approved by the Institutional Review Board of Third Xiangya Hospital, Central South University (No. 20040).

All KTRs received kidney transplantation from donation after citizen death (DCD) after 2012 or from close family members. The allografts from DCD were attributed by the China Organ Transplant Response System. All transplants performed were approved by the Ethics Committee of the Third Xiangya Hospital, Central South University. Routine induction therapy included anti-thymocyte globulin (ATG, 1.00 mg/kg daily for 3 days) or basiliximab (20 mg at Days 0 and 4), and the standard triple immunosuppressive regimen, namely, calcineurin inhibitor (CNI), mycophenolate mofetil/enteric-coated mycophenolate sodium, and corticosteroid was given as a maintenance regimen.

### Assessment of the Severity of Pneumonia

The KTRs diagnosed with pneumonia were divided into the severe pneumonia subgroup and the non-severe pneumonia subgroup according to the IDSA/ATS criteria for severe CAP, which include two major criteria and nine minor criteria (10). Severe pneumonia was defined as patients who met one or more major criteria, or three or more minor criteria. To further quantify the severity of pneumonia, the SOFA score and the IDSA/ATS minor criteria were used, which were reported to have the best performance in operationalization of pneumonia severity (20).

### Flow Cytometry

Peripheral blood mononuclear cells (PBMCs) were freshly isolated from EDTA-anticoagulated peripheral blood by density gradient centrifugation (Histopaque-1077, Sigma–Aldrich, 1.077 g/ml), which included low-density granulocytes (LDGs). After staining with live/dead dye (Zombie Aqua, Biolegend, USA), PBMCs were stained with the surface antibody cocktail for MDSC analysis: CD45-PE (clone HI30, eBioscience, San Diego, CA, USA), CD11b-Alexa Fluor 488 (clone ICRF44, Invitrogen), HLA-DR-APC (clone LN3, eBioscience), CD15-PerCP-eFluor 710 (clone MMA, eBioscience), and CD14-Super Bright 600 (clone 61D3, eBioscience). G-MDSCs were defined as CD11b^+^CD14^−^CD15^+^ LDGs, and M-MDSCs were defined as CD11b^+^CD14^+^HLA-DR^low/−^CD15^−^ cells. The detailed gating strategy is shown in Supplementary Figure 1. Flow cytometry was performed using BD FACSCanto II (BD Biosciences, NJ, USA). Data analysis was carried out with FlowJo 10.4 software.

### MDSC Effector Analysis

The representative effectors of MDSCs, such as arginase-1 (Arg-1), the S100 family of calcium binding proteins S100A8/A9 and S100A12, were analyzed in the plasma. The plasma samples were collected and stored at −80°C before detection. Arg-1, S100A8/A9, and S100A12 were detected using ELISA kits (ab230930, ab267628, and ab213822 Abcam, Cambridge, UK) on an ELx808IU microplate reader (BioTex, Houston, TX, USA) according to the instructions of manufacturer.

### Statistical Analysis

Continuous data are presented as the mean ± *SD* or median with interquartile range (IQR) and were compared using Student's *t*-test, Welch's *t*-test or the Mann–Whitney *U*-test, where appropriate. Categorical data were compared using Pearson's chi-squared (χ^2^) test or Fisher's exact test, where appropriate. Correlation analysis was performed using Spearman's rank correlation coefficient. The comparison for the repeated measurements of MDSCs was performed using a linear mixed model. Statistical analysis was performed using SPSS version 22.0 (SPSS, Inc., Chicago, IL, USA). *P* < 0.05 was considered to be statistically significant.

## Results

### Population Characteristics

A total of 90 KTRs suspected of pneumonia with 179 MDSC tests were first enrolled, but 32 KTRs with 50 MDSC tests were then excluded according to the diagnostic criteria of pneumonia. Among the remaining 58 KTRs, 18 patients were classified into the severe pneumonia subgroup based on the IDSA/ATS criteria for severe CAP. Thirteen of the 18 severe patients finished three MDSC tests, but four patients underwent only two MDSC tests, while one patient underwent one MDSC test due to their limited hospital stay. Similarly, 40 patients with non-severe pneumonia underwent 81 MDSC tests. The study flow and criteria are shown in Figure 1.

Another 29 stable KTRs without infection or tumor were also recruited as a control at a ratio of 2:1 and received the MDSC test once. The basic characteristics of the populations are shown in Table 1 and the corticosteroid usage is shown in Supplementary Table 1. The sex, age, donor source, CNI regimen, maintenance dosage of corticosteroids, and time from transplant to the first MDSC test showed no significant differences between the pneumonia group and the stable group. The severe pneumonia subgroup and the non-severe pneumonia subgroup also showed no significant difference in sex, age, donor source, or CNI regimen, but the time from transplant to the first MDSC test, namely, the onset time of pneumonia from transplant, was much shorter in the severe pneumonia subgroup (7.0 ± 6.0 vs. 30.0 ± 29.3 months, *p* < 0.001). In the severe pneumonia subgroup, 10 patients (55.6%) received pulse corticosteroid therapy, while none of patients in the non-severe pneumonia subgroup received pulse therapy. In contrast, the majority of patients (80.0%) in the non-severe pneumonia subgroup maintained a low dose of corticosteroids. The parameters that described the severity of pneumonia, such as the SOFA score and the IDSA/ATS minor criteria, were also significantly higher in the severe pneumonia subgroup (SOFA score 3.3 ± 1.5 vs. 1.1 ± 0.9, *p* < 0.001; IDSA/ATS minor criteria 3.1 ± 0.6 vs. 1.3 ± 0.8, *p* < 0.001). One patient in the severe pneumonia subgroup lost allograft function during treatment but recovered from pneumonia, and another patient in the severe pneumonia subgroup died of pneumonia with a functioning allograft.

**Table 1:** Characteristics of the study population.

	**All patients**	**Pneumonia patients**, ***n*** **=** **58**			**Stable patients**	***P*-value^#^**
	***n* = 87**	**Severe pneumonia *n* = 18**	**Nonsevere pneumonia *n* = 40**	*P*-value^*^	***n* = 29**	
Male recipient, *n* (%)	55 (63.2%)	10 (55.6%)	24 (60.0%)	0.751	21 (71.4%)	0.208
34 (58.6%)						
Age, yrs ± SD	43.3 ± 10.9	38.8 ± 12.9	43.3 ± 10.5	0.173	46.3 ± 9.3	0.075
41.9 ± 11.4						
Donor, *n* (%)						0.155^§^
DCD	77 (88.5%)	17 (94.4%)	32 (80.0%)	0.249^§^	28 (96.6%)	
49 (84.5%)						
Relative	10 (11.5%)	1 (5.6%)	8 (20.0%)		1 (3.4%)	
9 (15.5%)						
Calcineurin inhibitor, *n* (%)						0.296^§^
Tacrolimus	83 (95.4%)	15 (83.3%)	39 (97.5%)	0.084^§^	29 (100.0%)	
54 (93.1%)						
Cyclosporine A	4 (4.6%)	3 (16.7%)	1 (2.5%)		0 (0.0%)	
4 (6.9%)						
Time from transplant to 1^st^ MDSC test, months ± SD	21.7 ± 21.9	7.0 ± 6.0	30.0 ± 29.3	<0.001^†^	19.4 ± 3.6	0.332^†^
22.9 ± 26.8						
Hospital length of stay, d	–	26.2 ± 17.0	11.5 ± 7.3	0.002^†^	–	–
16.0 ± 13.0						
MDSC (%PBMC, median with interquartile range)	18.2 (11.2, 26.8)	28.8 (18.9, 42.7)	20.2 (16.2, 26.8)	0.033^¶^	10.6 (9.4, 15.4)	<0.001^¶^
21.7 (16.6, 31.6)						
G-MDSC (%PBMC, median with interquartile range)	6.2 (3.0, 11.1)	9.3 (6.8, 27.3)	4.2 (2.5, 8.4)	0.002^¶^	5.4 (2.8, 10.1)	0.715^¶^
6.5 (3.2, 12.0)						
M-MDSC (%PBMC, median with interquartile range)	8.8 (4.8, 16.7)	12.4 (3.5, 23.3)	15.4 (9.5, 19.7)	0.410^¶^	5.1 (4.3, 6.1)	<0.001^¶^
14.0 (8.2, 19.9)						
Argnase-1, ng/ml (median with interquartile range)	1.8 (1.2, 3.7)	1.5 (0.9, 2.6)	2.0 (1.5, 5.2)	0.060^¶^	1.0 (0.6, 2.3)	0.014^¶^
1.9 (1.4, 4.1)						
S100A8/A9, ng/ml (median with interquartile range)	2263.0 (961.9, 5425.3)	5277.0 (2682.7, 12263.4)	2295.5 (1235.8, 5385.2)	0.023^¶^	868.2 (741.4, 1557.1)	0.001^¶^
3669.9 (1486.4, 6295.4)						
S100A12, ng/ml (median with interquartile range)	117.3 (46.2, 408.5)	927.2 (274.7, 2770.3)	120.6 (67.7, 263.7)	<0.001^¶^	0.007 (0.002, 0.013)	<0.001^¶^
171.7 (77.7, 704.1)						
SOFA	–	3.3 ± 1.5	1.1 ± 0.9	<0.001^†^	–	–
1.8 ± 1.5						
IDSA/ATS minor criteria	–	3.1 ± 0.6	1.3 ± 0.8	<0.001	–	–
1.8 ± 1.1						
Graft loss, *n* (%)	–	1 (5.6%)	0 (0.0%)	0.310^§^	–	–
1 (1.7%)						
Death, *n* (%)	–	1 (5.6%)	0 (0.0%)	0.310^§^	–	–
1 (1.7%)						

* *Comparison between the severe pneumonia patients and the non-severe pneumonia patients*.

# *Comparison between the pneumonia patients and the stable patients*.

§ *Tested by Fisher's exact test*.

¶ *Tested by Mann-Whitney U test*.

† *Tested by Welch's t-test*.

*SD, standard deviation; DCD, donation after citizens' death; MDSC, myeloid-derived suppressor cells; %PBMC, percentage of peripheral blood mononuclear cells; G-MDSC, granulocytic MDSC; M-MDSC, monocytic MDSC; SOFA, sequential organ failure assessment; IDSA, infectious diseases society of america; ATS, american thoracic society*.

### Increased MDSCs and Effectors in KTRs With Pneumonia

**Figure 2:**
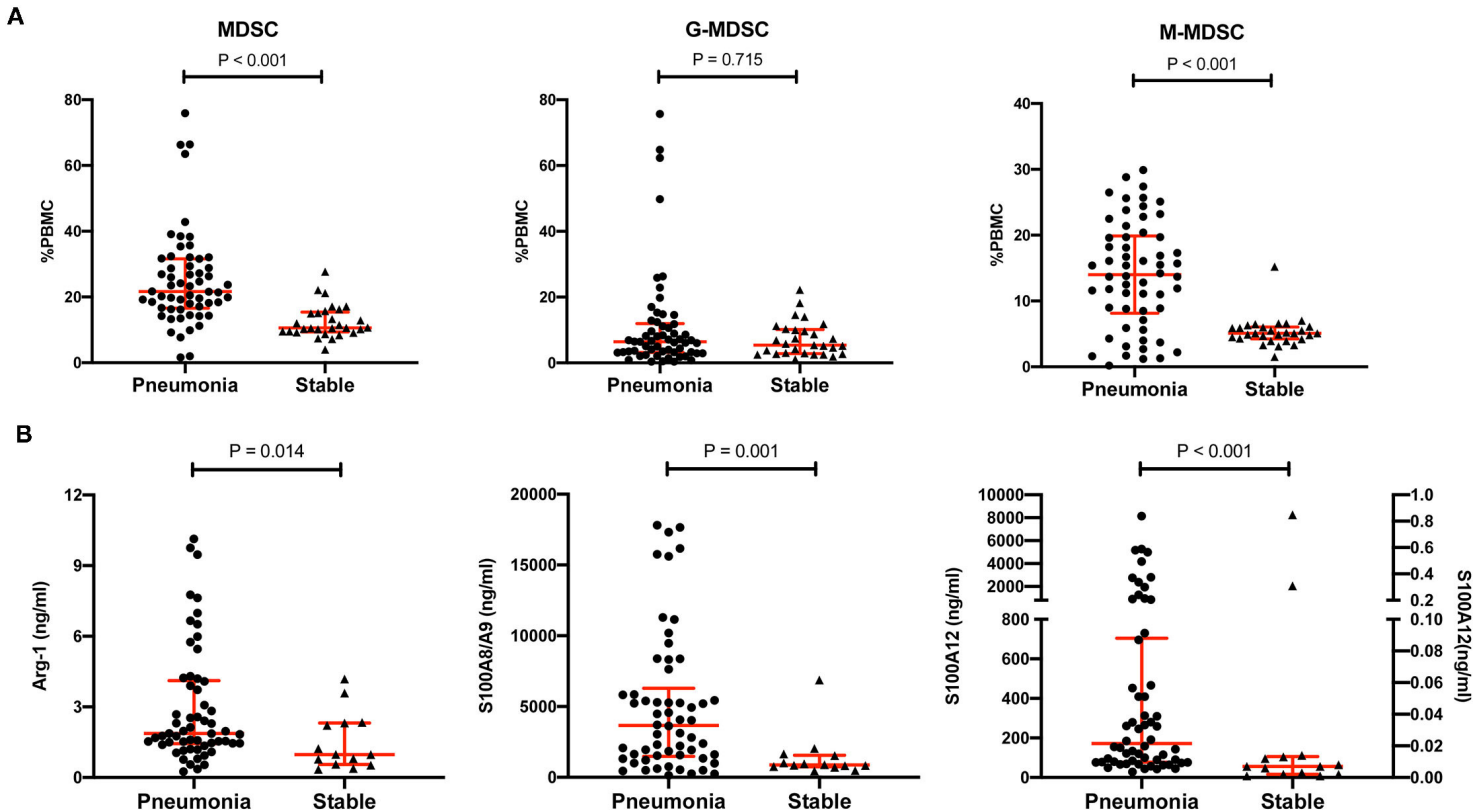
Comparison of MDSCs, MDSC subsets, and MDSC effectors between the pneumonia group and the stable group. **(A)** The percentages of MDSCs, CD11b^+^CD14^−^CD15^+^ LDG G-MDSCs and CD11b^+^CD14^+^HLA-DR^low/−^CD15^−^ M-MDSCs in peripheral blood mononuclear cells (PBMCs) in the pneumonia group (*n* = 58) and the stable group (*n* = 29). Tested by the Mann–Whitney *U* test. **(B)** MDSC effectors, such as Arg-1, S100A8/A9 and S100A12, in the pneumonia group (*n* = 58) and the stable group (*n* = 14). Tested by the Mann–Whitney *U* test. For S100A12, the left Y axis represents the pneumonia group and the right Y axis represents the stable group. MDSC, myeloid-derived suppressor cell; LDG, low-density granulocyte; G-MDSC, granulocytic-MDSC; M-MDSC, monocytic-MDSC; PBMCs, peripheral blood mononuclear cells; Arg-1, arginase-1.

The percentage of MDSCs in PBMCs and the representative effectors, including Arg-1, S100A8/A9 and S100A12, were compared between the pneumonia group and the stable group. The data of the first MDSC test in the pneumonia group were used for comparison. As shown in Table 1 and Figure 2, the MDSCs were much higher in the pneumonia group than in the stable group (21.7%, IQR 16.6–31.6% vs. 10.6%, IQR 9.4–15.4%, *p* < 0.001). For the subsets of MDSCs, the M-MDSC subset was much higher in the pneumonia group (14.0%, IQR 8.2–19.9% vs. 5.1%, IQR 4.3–6.1%, *p* < 0.001), but the G-MDSC subset showed no significant difference (6.5%, IQR 3.2–12.0% vs. 5.4%, IQR 2.8–10.1%, *p* = 0.715). All the MDSC effectors detected were much higher in the pneumonia group, especially S100A12, which showed an order of magnitude difference (171.7, IQR 77.7–704.1 vs. 0.007, IQR 0.002–0.013, *p* < 0.001).

### G-MDSCs Correlated With the Severity of Pneumonia

**Figure 3:**
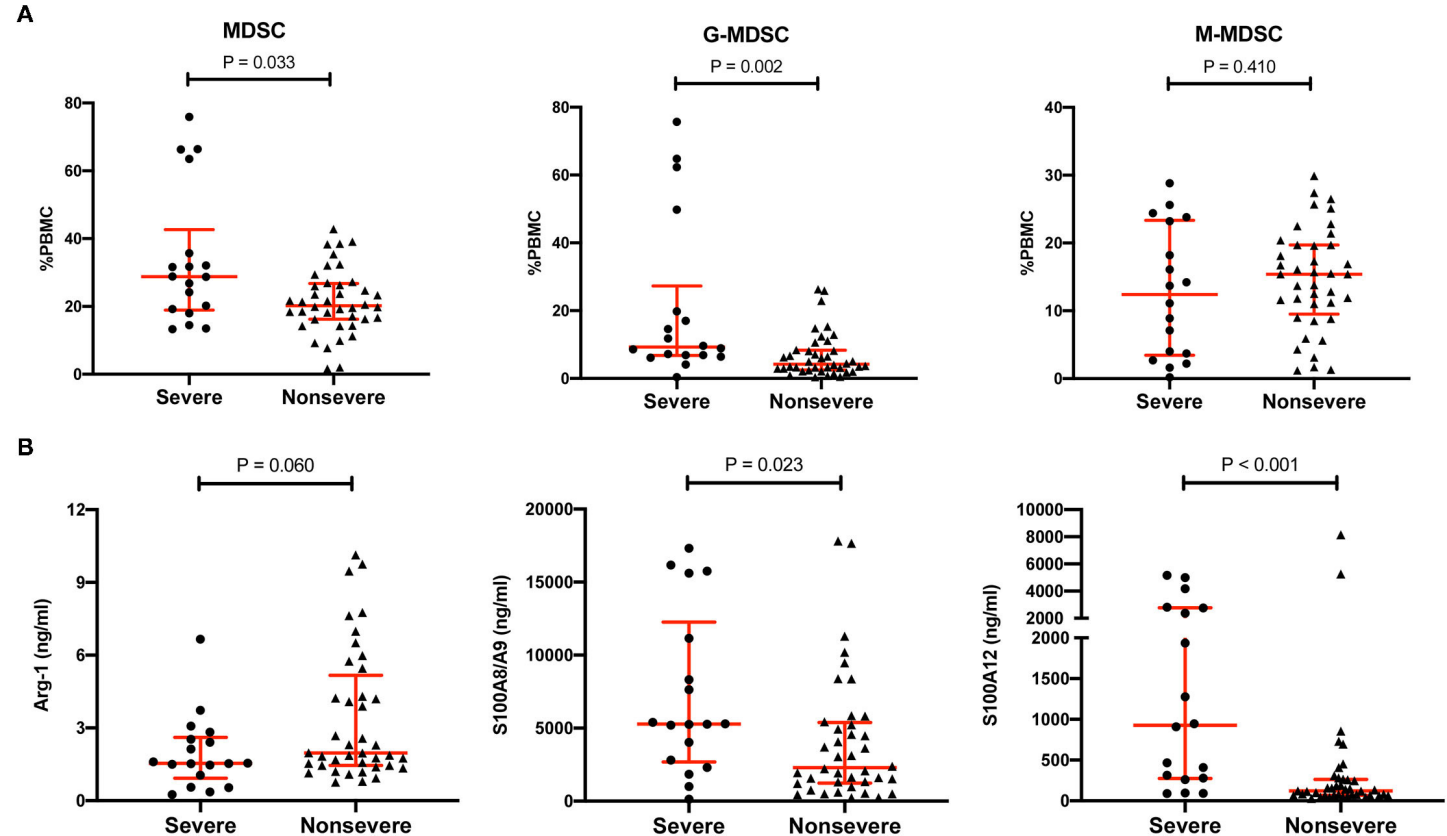
Comparison of MDSCs, MDSC subsets, and MDSC effectors between the severe pneumonia subgroup and the non-severe pneumonia subgroup. **(A)** The percentages of MDSCs, CD11b^+^CD14^−^CD15^+^ LDG G-MDSCs and CD11b^+^CD14^+^HLA-DR^low/−^CD15^−^ M-MDSCs in PBMCs in the severe pneumonia subgroup (*n* = 18) and the non-severe pneumonia subgroup (*n* = 40). Tested by the Mann–Whitney *U* test. **(B)** MDSC effectors, such as Arg-1, S100A8/A9, and S100A12, in the severe pneumonia subgroup (*n* = 18) and the non-severe pneumonia subgroup (*n* = 40). Tested by the Mann–Whitney *U* test. MDSC, myeloid-derived suppressor cell; LDG, low-density granulocyte; G-MDSC, granulocytic-MDSC; M-MDSC, monocytic-MDSC; PBMCs, peripheral blood mononuclear cells; Arg-1, arginase-1.

To further explore the correlation between MDSCs and pneumonia in KTRs, MDSCs and their effectors were compared between the severe subgroup and the non-severe subgroup (Figure 3). The MDSCs were also higher in the severe pneumonia subgroup than in the non-severe pneumonia group (28.8%, IQR 18.9–42.7% vs. 20.2%, IQR 16.2–26.8%, *p* = 0.033). Notably, the M-MDSC subset was maintained a high level in both subgroups and showed no significant difference (12.4%, IQR 3.5–23.3% vs. 15.4%, IQR 9.5–19.7%, *p* = 0.410). In contrast, the G-MDSC subset was much higher in the severe pneumonia subgroup (9.3%, IQR 6.8–27.3% vs. 4.2%, IQR 2.5–8.4%, *p* = 0.002). S100A8/A9 and S100A12 were also higher in the severe subgroup, but Arg-1 was similar between the two subgroups.

**Figure 4:**
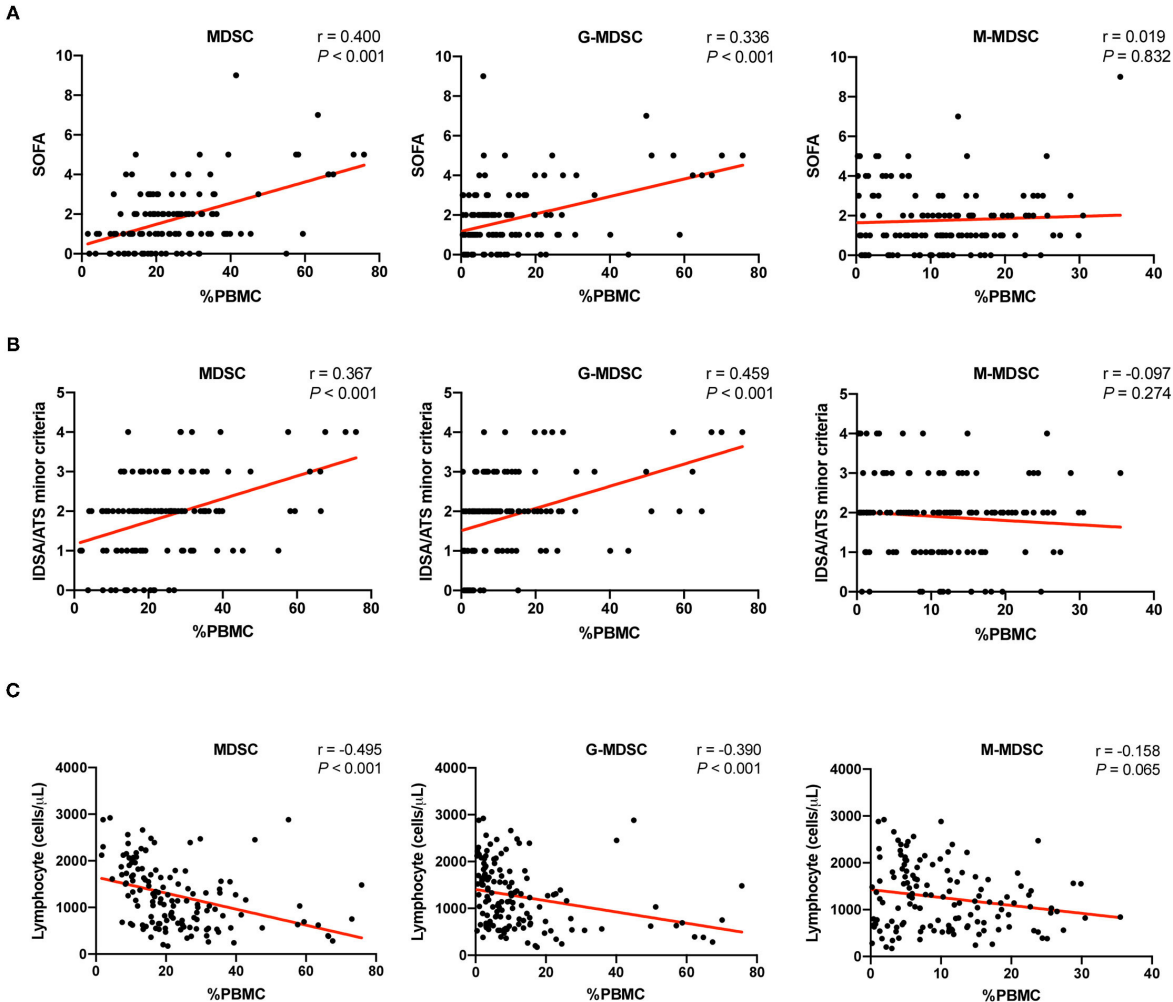
The correlation between MDSCs and the severity of pneumonia as well as lymphopenia. **(A)** The correlation between the percentages of MDSCs and MDSC subsets in PBMCs and the severity of pneumonia, which was assessed by the sequential organ failure assessment (SOFA) score. The MDSC results and the corresponding SOFA scores of the patients at different time points were included (*n* = 129). Tested by the Spearman's rank correlation coefficient. **(B)** The correlation between the percentages of MDSCs and MDSC subsets in PBMCs and the severity of pneumonia, which was assessed by the IDSA/ATS minor criteria. The MDSC results and the corresponding IDSA/ATS minor criteria scores of the patients at different time points were included (*n* = 129). Tested by the Spearman's rank correlation coefficient. **(C)** The correlation between the percentages of MDSCs and MDSC subsets in PBMCs and the cell counts of lymphocytes. The MDSC results and the corresponding lymphocyte counts of the patients at different time points were included (*n* = 136, such as stable patients). Tested by the Spearman's rank correlation coefficient. MDSC, myeloid-derived suppressor cell; G-MDSC, granulocytic-MDSC; M-MDSC, monocytic-MDSC; PBMCs, peripheral blood mononuclear cells; SOFA, sequential organ failure assessment; IDSA/ATS, infectious diseases society of america/american thoracic society.

Two scoring systems, namely, or including the SOFA score and the IDSA/ATS minor criteria were used as operationalized parameters to quantify the severity of pneumonia as previously reported (20). Correlation analysis between these scores and the corresponding MDSCs was performed with all the data. As shown in Figure 4, both MDSCs and G-MDSCs were correlated with the severity of pneumonia, while M-MDSCs were not. Among all criteria measured, G-MDSCs had the strongest correlation with the IDSA/ATS minor criteria (*r* = 0.459, *p* < 0.001).

An important feature of severe pneumonia is immunosuppression, which manifests as lymphopenia. Correlation analysis between the lymphocyte counts and the corresponding MDSCs was also performed. Similarly, MDSCs and G-MDSCs were negatively correlated with lymphocyte counts, while M-MDSCs had no significant correlation (Figure 4).

### Severe Pneumonia KTRs Maintained Consistently High Levels of MDSCs and G-MDSCs

**Figure 5:**
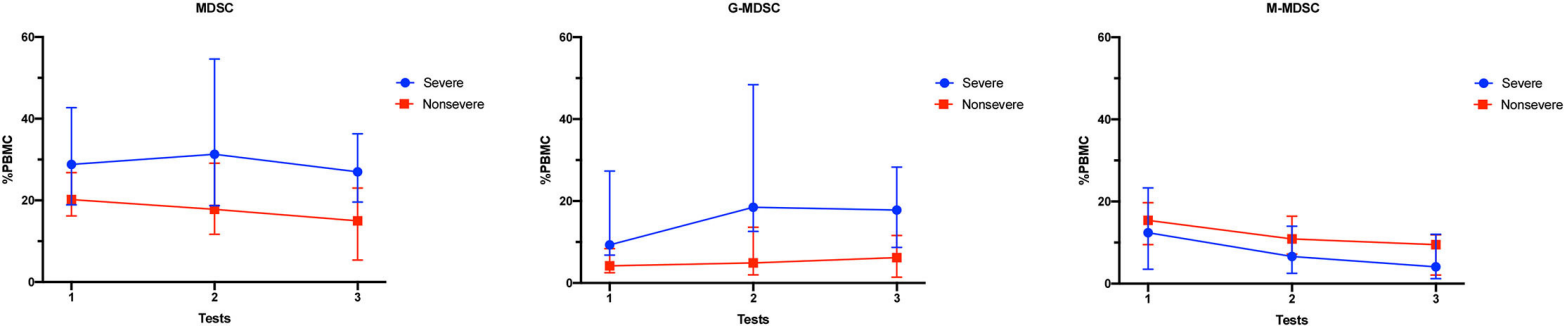
Longitudinal MDSC frequencies in pneumonia kidney transplant recipients. The percentages of MDSCs and MDSC subsets in PBMCs of the severe pneumonia subgroup and the non-severe pneumonia subgroup at different time points. The results are shown as medians with interquartile ranges. MDSC, myeloid-derived suppressor cell; M-MDSC, monocytic-MDSC; G-MDSC, granulocytic-MDSC; PBMCs, peripheral blood mononuclear cells.

The longitudinal MDSC results of the severe and non-severe pneumonia subgroups at different time points are shown in Figure 5 and Table 2. The linear mixed model was used for analysis, and the results are shown in Table 3. Both subgroups showed sustained high levels of MDSCs, which did not decrease during the test period (time as the factor, *p* = 0.285). For the subsets of MDSCs, the G-MDSCs were maintained at a high level (*p* = 0.356), while the M-MDSCs decreased over time (*p* = 0.021). The severe pneumonia subgroup manifested consistently higher levels of MDSCs and G-MDSCs than the non-severe pneumonia subgroup (severity as the factor, both *p* < 0.001), but the M-MDSCs showed no significant difference between the two subgroups (*p* = 0.345). No significant interaction effect between time and severity was seen in the analysis (all *p* > 0.05).

**Table 2:** MDSCs of pneumonia patients tested at different time points.

	**Pneumonia patients**			**Severe pneumonia patients**			**Nonsevere pneumonia patients**		
	**1st, *n* = 58**	**2nd, *n* = 49**	**3rd, *n* = 22**	**1st, *n* = 18**	**2nd, *n* = 16**	**3rd, *n* = 14**	**1st, *n* = 40**	**2nd, *n* = 33**	**3rd, *n* = 8**
MDSC (%PBMC, median with interquartile range)	21.7 (16.6, 31.6)	22.0 (14.4, 34.4)	23.0 (12.7, 31.0)	28.8 (18.9, 42.7)	31.3 (18.7, 54.6)	27.0 (19.6, 36.3)	20.2 (16.2, 26.8)	17.8 (11.7, 29.1)	15.0 (5.4, 23.0)
G-MDSC (%PBMC, median with interquartile range)	6.5 (3.2, 12.0)	10.0 (3.0, 21.0)	12.0 (5.3, 24.9)	9.3 (6.8, 27.3)	18.5 (12.6, 48.4)	17.8 (8.7, 28.3)	4.2 (2.5, 8.4)	4.9 (2.0, 13.6)	6.2 (1.4, 11.6)
M-MDSC (%PBMC, median with interquartile range)	14.0 (8.2, 19.9)	10.0 (5.7, 15.1)	6.3 (1.3, 11.7)	12.4 (3.5, 23.3)	6.6 (2.5, 14.0)	4.1 (1.2, 12.0)	15.4 (9.5, 19.7)	10.9 (7.2, 16.4)	9.5 (2.1, 11.9)

*MDSC, myeloid-derived suppressor cells; %PBMC, percentage of peripheral blood mononuclear cells; G-MDSC, granulocytic MDSC; M-MDSC, monocytic MDSC*.

**Table 3:** The comparison of MDSCs and MDSC subsets between severe and nonsevere pneumonia patients at different time points.

	**MDSC**		**G-MDSC**		**M-MDSC**	
	***F* value**	***P*-value**	***F* value**	***P*-value**	***F* value**	***P*-value**
Intercept	347.710	<0.001	115.335	<0.001	162.826	<0.001
Severity	23.762	<0.001	27.778	<0.001	0.908	0.345
Time	1.292	0.285	1.058	0.356	4.240	0.021
Severity * Time	0.076	0.927	0.270	0.765	0.440	0.647

*Tested by linear mixed model analysis. MDSC, myeloid-derived suppressor cells; G-MDSC, granulocytic MDSC; M-MDSC, monocytic MDSC*.

## Discussion

Although great effort has been put into prophylaxis against infections post-kidney transplantation, infectious diseases are still one of the leading causes of death among KTRs. Reportedly, the cumulative incidence of post-transplant infection was as high as 36.9% at 3 months, 53.7% at 1 year, 69.6% at 3 years, and 78.0% at 5 years, which dramatically increased the mortality rate and DCGF risk of KTRs and additionally places an extra burden on the healthcare insurance system (4). Pneumonia is one of the most common but serious infectious complications post-kidney transplantation. Severe pneumonia can progress into acute respiratory distress syndrome, sepsis, shock, and multiple organ failure and can eventually lead to death. Kinnunen et al. reported that pulmonary infection defined as bacterial or unspecified pneumonia accounted for 45% of all infection-related deaths among KTRs and was the most frequent cause of death (7). Compared with CAP in the general population, pneumonia in KTRs is challenging due to various clinical presentations, numerous potential etiologies, and rapid changes in condition.

A critical feature of pneumonia in KTRs is that patients are immunocompromised; therefore, conventional CAP severity scores, such as PSI and CURB-65, without assessment of the state of immunosuppression, are not recommended for KTRs (21). Indeed, no pneumonia severity score is currently available for KTRs or patients after solid organ transplantation. Instead, a two-tier approach was proposed by the American Society of Transplantation Infectious Diseases Community of Practice, which is based on the net state of immunosuppression and the severity of presentation (21). Nevertheless, this approach is not quantitatively capable of assessing the severity of pneumonia. A recent large-scale multicentric observational study, the PROGRESS study, compared a series of scoring systems to find a valid, reproducible, and quantitative measure to describe CAP severity (20). The results showed that the SOFA score had the best ability to identify a severe state of CAP, followed by the IDSA/ATS minor criteria (20). Therefore, these two scoring systems, which contained parameters, such as leukopenia and thrombocytopenia to assess the immune state, were adopted in this study to describe the severity of pneumonia in KTRs. Both scores showed a significant difference between the severe pneumonia subgroup and the non-severe pneumonia subgroup, indicating the possible applicability of these scores in KTRs. However, our study indicates that the parameter assessing renal function in these scores is too strict for KTRs and may need to be relaxed for KTRs and validated in a larger cohort.

As the guideline recommends, assessment of the net state of immunosuppression is critical to evaluate the severity of pneumonia in KTRs (21). If the patient is in a state of severe immunosuppression, measures, such as immunosuppressant reduction or even withdrawal should be taken. The key question is: How can we accurately assess the immune status of the patient? Any inappropriate treatment strategy may lead to serious consequences, such as allograft rejection or aggravation of pneumonia. The conventional methods to assess immunosuppression include therapeutic drug monitoring and complete blood count with differential, but these results are too rough to accurately reflect the complex immune system (21, 22). Therefore, an increasing number of immune biomarkers are being introduced to solid organ transplantation for immune monitoring (8). The combination of certain immune biomarkers, as we have reported, shows better performance than any single parameter in this area (9).

MDSCs were first identified in a cancer-related context, and the term “myeloid-derived suppressor cell” indicated the myeloid origin and immunosuppressive function (23). In fact, in a variety of pathological conditions, such as cancer, infections, and tissue damage, steady-state myelopoiesis are switched to emergency myelopoiesis, and MDSCs are induced (13, 16, 24). Unlike mature granulocytes or monocytes, the key feature of MDSCs is immunosuppression, and a variety of mechanisms are involved (15). In sepsis, factors, such as growth signals (granulocyte-macrophage colony-stimulating factors [GM-CSF], granulocyte-CSF [G-CSF], macrophage-CSF [M-CSF], etc.), damage-associated molecular patterns (DAMPs, such as S100A8/A9, S100A12 and high mobility group box-1 protein), pathogen-associated molecular patterns (PAMPs, such as lipopolysaccharide and staphylococcal enterotoxins), and inflammatory cytokines [interleukin (IL)-1β, IL-6, interferon -γ, etc.] rise sharply at the early stage, which is called the “cytokine storm”, and MDSCs are induced to serve as a negative feedback loop to control hyperinflammation and prevent tissue injury and organ dysfunction (25). However, immunoparalysis is also induced in sepsis at the same time, which favors secondary infections and long-term immune disabilities. Some patients with sepsis survive the initial sepsis events but suffer from chronic critical illness (CCI), which is characterized by long-lasting immunosuppression associated with persistent, low-grade inflammation (25). The term persistent inflammation, immunosuppression, and catabolism syndrome (PICS) is proposed to illustrate the underlying mechanism of CCI, and persistently increased MDSCs are believed to play an important role in PICS (14, 26, 27). Mathias et al. reported that G-MDSCs were dramatically increased in the patients with sepsis/septic shock and persisted for 28 days. G-MDSCs expressed the *HLA* gene at a low level but upregulated *ARG1* gene expression and suppressed T-cell proliferation *in vitro*. More importantly, the patients with sepsis/septic shock with persistently high levels of MDSCs were associated with adverse outcomes, such as nosocomial infections and poor functional status (28). Uhel et al. also confirmed the specific expansion of G-MDSCs in sepsis patients, and a high initial G-MDSC level was associated with subsequent nosocomial infections (17).

The situation of pneumonia is similar to that of sepsis, especially in severe pneumonia. The novel coronavirus severe acute respiratory syndrome coronavirus 2 (SARS-CoV-2) global pandemic can cause severe pneumonia, which manifests hyperinflammation and immunosuppression (29). MDSCs were found to expand in the patients infected with SARS-CoV-2, and the frequency of MDSCs was correlated with COVID-19 disease severity (18, 19). Therefore, the frequency of MDSCs was suggested as a predictor of COVID-19 severity. In this study, we focused on pneumonia in KTRs, an immunocompromised population. We confirmed the persistently increased MDSCs in patients with pneumonia and further found that G-MDSCs were strongly correlated with the severity of pneumonia. The results were in accordance with the previous studies, which revealed that G-MDSCs were specifically correlated with the disease severity (17, 18, 28). Therefore, the frequency of G-MDSCs could be an indicator of pneumonia severity in KTRs. One possible explanation was that significantly higher DAMPs, such as S100A8/A9 and S100A12, were detected in this study, or PAMPs were released in patients with more severe conditions, which intensively promoted emergency granulopoiesis and induced G-MDSCs (13). There was a difference in M-MDSCs between the patients with stable and pneumonia, but the frequency of M-MDSCs was not correlated with disease severity (17).

Serious immunosuppression was seen in KTRs with severe pneumonia, even though these patients stopped immunosuppressants, such as CNI. Indeed, eight of the 18 patients with severe pneumonia in this study stopped CNI and mycophenolic acid and only received intermittent steroids. However, severe lymphopenia and/or leukopenia were sustained, which was in accordance with the manifestations of PICS. We found that the lymphocyte count was negatively correlated with the frequency of MDSCs, especially G-MDSCs, suggesting that G-MDSCs contributed to immunosuppression in these patients.

A critical obstacle for the clinical application of MDSCs as biomarkers for immune monitoring is the complexity and ambiguity of these heterogeneous cells. In mice, the phenotype of MDSCs is relatively definite: CD11b^+^Ly6G^+^Ly6C^low^ G-MDSCs and CD11b^+^Ly6G^−^Ly6C^hi^ M-MDSCs. However, for human MDSCs, it is difficult to define heterogeneous populations of MDSCs using definite cell surface markers. Some scholars recommended defining human G-MDSCs as CD11b^+^CD14^−^CD15^+^ (or CD66b^+^) LDGs, M-MDSCs as CD11b^+^CD14^+^CD15^−^HLA-DR^low/−^ and other more immature progenitors, that is, the “earlystage MDSCs” (eMDSCs) as Lin^−^(CD3/14/15/19/56) HLA-DR^−^CD33^+^ (16). We used this definition in this study, but in fact, the nomenclature and characterization standard of MDSCs varied in different studies, which made it difficult to compare the results horizontally. Some novel markers have been reported to define MDSCs, such as S100A9 for M-MDSCs (30) and lectin-type oxidized low-density lipoprotein receptor 1 (LOX-1) for G-MDSCs (31), but more studies are still needed to further define the identity and nature of MDSCs.

There were some limitations in this study. The sample size was limited, and patients were recruited at a single institution. MDSCs were detected at only three points over 2 weeks, and some patients did not complete all tests because they were discharged. Due to clinical constraints, only the phenotypes and effectors of MDSCs were detected, and *in vitro* function assay was not performed.

In conclusion, we found that MDSCs and G-MDSCs were persistently increased in KTRs with pneumonia. Parameters concerning immune status should be added to assess the severity of pneumonia for KTRs. G-MDSCs were correlated with the severity of pneumonia and could thus serve as an indicator for assessing pneumonia severity.

## Data Availability Statement

The original contributions presented in the study are included in the article/Supplementary Material, further inquiries can be directed to the corresponding author.

## Ethics Statement

The studies involving human participants were reviewed and approved by the Institutional Review Board of Third Xiangya Hospital, Central South University. The patients/participants provided their written informed consent to participate in this study.

## Author Contributions

BP, YL, and YM conceived and designed the study. BP, YL, and MY performed the MDSC tests and collected the data. PZ, YZ, and GK performed the ELISA tests. QZ, JL, and KC provided expert advice and assisted data analysis. BP analyzed the data and wrote the manuscript. KC and YM revised the manuscript. All authors read and approved the final manuscript.

## Funding

This study was supported by the National Natural Science Foundation of China (81771722) and the Hunan Provincial Natural Science Foundation (2020JJ5863).

## Conflict of Interest

The authors declare that the research was conducted in the absence of any commercial or financial relationships that could be construed as a potential conflict of interest.

## Publisher's Note

All claims expressed in this article are solely those of the authors and do not necessarily represent those of their affiliated organizations, or those of the publisher, the editors and the reviewers. Any product that may be evaluated in this article, or claim that may be made by its manufacturer, is not guaranteed or endorsed by the publisher.
