# Supplementary material for: The Expansion of Myeloid-Derived Suppressor Cells Correlates With the Severity of Pneumonia in Kidney Transplant Patients
Source: Front Med (Lausanne). 2022 Feb 15;9:795392. doi: 10.3389/fmed.2022.795392 (PMC8885803; doi:10.3389/fmed.2022.795392)
Supplement: Supplementary file 1 [file Data_Sheet_1.DOCX]

Supplementary Material

# Supplementary Figure


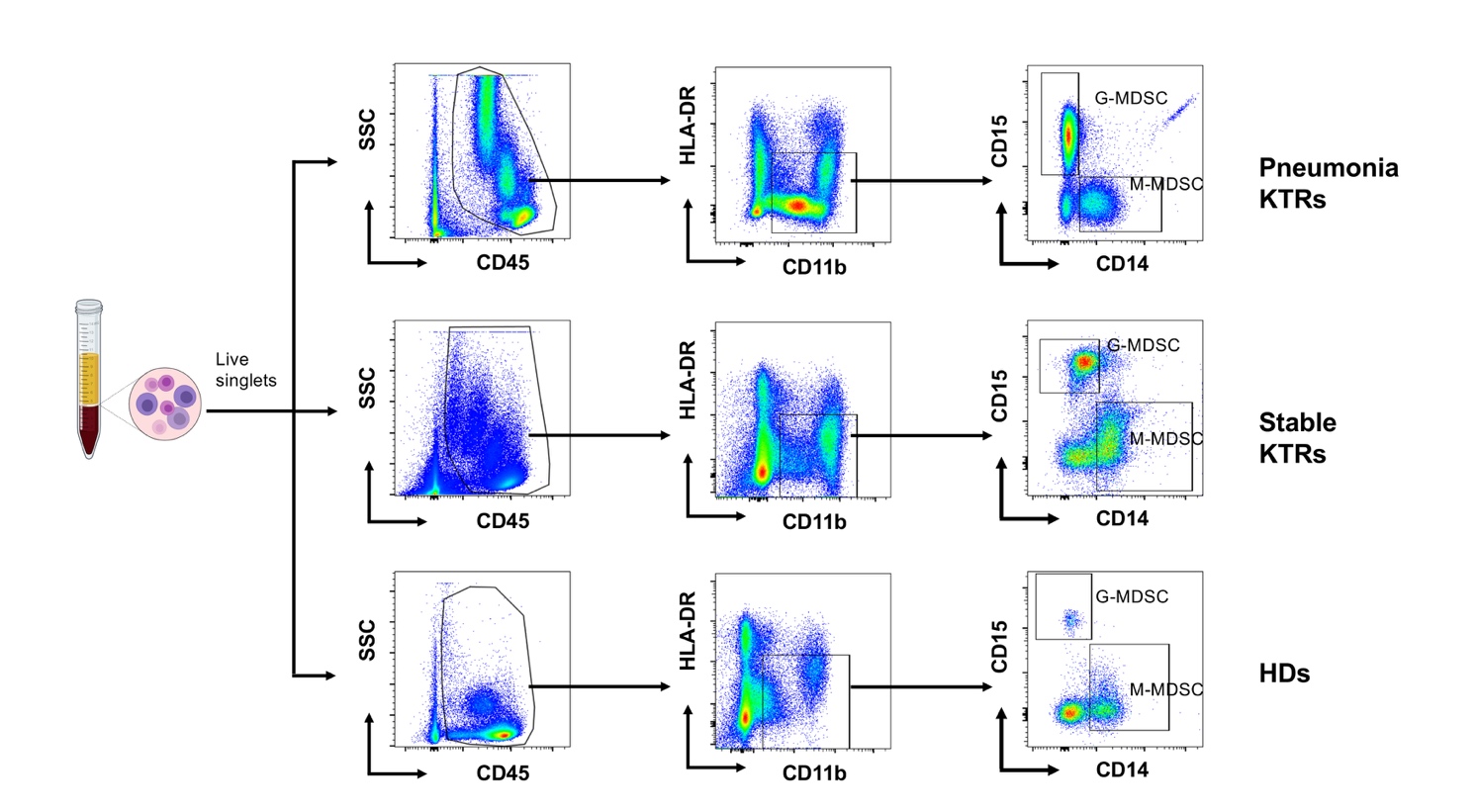


**Supplementary Figure 1.** The protocol and gating strategy of MDSCs. PBMCs were freshly isolated from the peripheral blood by density gradient centrifugation and stained with the surface marker cocktail. After removing dead cells and cell clots, G-MDSCs were defined as CD11b^+^CD14^-^ HLA-DR^-^CD15^+^ cells and M-MDSCs were defined as CD11b^+^CD14^+^HLA-DR^low/-^CD15^-^ cells. MDSC, myeloid-derived suppressor cell. PBMC, peripheral blood mononuclear cell. M-MDSC, monocytic-MDSC. G-MDSC, granulocytic-MDSC. HDs, healthy donors.

# Supplementary Table

| **Supplementary table 1. Corticosteroid usage of the study population** | | | | | | |  |
| --- | --- | --- | --- | --- | --- | --- | --- |
|  | **All patients n = 87** | **Pneumonia patients, n = 58** | | | **Stable patients n = 29** | ***P* value^#^** | |
|  |  | **Severe pneumonia n = 18** | **Nonsevere pneumonia n = 40** | ***P* value^*^** |  |  |  |
| Maintenance dosage of corticosteroids, n (%) |  |  |  | 0.103 |  | 0.516 | |
| 2.5 mg prednisone or 2 mg methylprednisolone | 4 (4.6%) | 0 (0.0%) | 3 (7.5%) |  | 1 (3.4%) |  | |
|  |  | 3 (5.2%) | | |  |  | |
| 5 mg prednisone or 4 mg methylprednisolone | 79 (90.8%) | 15 (83.3%) | 36 (90.0%) |  | 28 (96.6%) |  | |
|  |  | 51 (87.9%) | | |  |  | |
| 10 mg prednisone or 8 mg methylprednisolone | 2 (2.3%) | 2 (11.1%) | 0 (0.0%) |  | 0 (0.0%) |  | |
|  |  | 2 (3.4%) | | |  |  | |
| over 10 mg prednisone or 8 mg methylprednisolone | 2 (2.3%) | 1 (5.6%) | 1 (2.5%) |  | 0 (0.0%) |  | |
|  |  | 2 (3.4%) | | |  |  | |
| Corticosteroid dosage during hospitalization, n (%) |  |  |  | < 0.001 |  |  | |
| None | – | 1 (5.6%) | 8 (20.0%) |  | – |  | |
|  |  | 9 (15.5%) | | |  |  | |
| 5 – 10 mg prednisone or 4 – 8 mg methylprednisolone | – | 7 (38.9%) | 32 (80.0%) |  | – |  | |
|  |  | 39 (67.2%) | | |  |  | |
| Pulse therapy | – | 10 (55.6%) | 0 (0.0%) |  | – |  | |
|  |  | 10 (17.2%) | | |  |  | |
| ^*^ Comparison between the severe pneumonia patients and the non-severe pneumonia patients. ^#^ Comparison between the pneumonia patients and the stable patients. | | | | | | |  |
